# New coordination features; a bridging pyridine and the forced shortest non-covalent distance between two CO_3_^2–^ species†
†Electronic supplementary information (ESI) available: Mass Spectrometry and BVS analysis CCDC 996546–996548. For ESI and crystallographic data in CIF or other electronic format see DOI: 10.1039/c4sc02491e



**DOI:** 10.1039/c4sc02491e

**Published:** 2014-10-08

**Authors:** V. Velasco, D. Aguilà, L. A. Barrios, I. Borilovic, O. Roubeau, J. Ribas-Ariño, M. Fumanal, S. J. Teat, G. Aromí

**Affiliations:** a Departament de Química Inorgànica , Universitat de Barcelona , Diagonal 645 , 08028 Barcelona , Spain . Email: guillem.aromi@qi.ub.es ; Tel: +34 934039760; b Instituto de Ciencia de Materiales de Aragón (ICMA) , CSIC and Universidad de Zaragoza , Plaza San Francisco s/n , 50009 , Zaragoza , Spain; c Departament de Química Física and IQTCUB , Universitat de Barcelona , Diagonal 645 , 08028 Barcelona , Spain; d Advanced Light Source , Berkeley Laboratory , 1 Cyclotron Road , Berkeley , California 94720 , USA

## Abstract

The aerobic reaction of the multidentate ligand 2,6-bis-(3-oxo-3-(2-hydroxyphenyl)-propionyl)-pyridine, H_4_L, with Co(ii) salts in strong basic conditions produces the clusters [Co_4_(L)_2_(OH)(py)_7_]NO_3_ (**1**) and [Co_8_Na_4_(L)_4_(OH)_2_(CO_3_)_2_(py)_10_](BF_4_)_2_ (**2**). Analysis of their structure unveils unusual coordination features including a very rare bridging pyridine ligand or two trapped carbonate anions within one coordination cage, forced to stay at an extremely close distance (*d*_O···O_ = 1.946 Å). This unprecedented non-bonding proximity represents a meeting point between long covalent interactions and “intermolecular” contacts. These original motifs have been analysed here through DFT calculations, which have yielded interaction energies and the reduced repulsion energy experimented by both CO_3_^2–^ anions when located in close proximity inside the coordination cage.

## Abstract 2

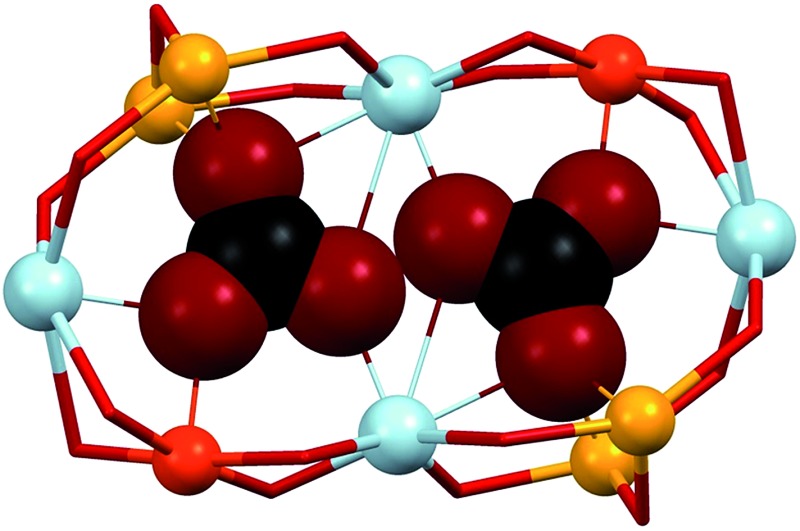
New coordination assemblies furnish a rare crevice pyridine ligand or encapsulation of the closest not bonded CO_3_^2–^ species.

## 1. Introduction

**Scheme 1:**
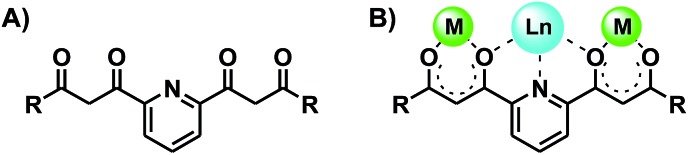
Pyridine-spaced bis-β-diketone ligands (A), and coordination mode in complexes of the type [M–Ln–M]^7+^ (B).

The coordination chemistry of 1,3-dicarbonyl-based multidentate ligands constitutes now an important subarea of structural molecular chemistry.^1–4^ The good chelating ability of β-diketonates together with a particular distribution throughout a given organic scaffold, in combination or not with additional donor groups has led to novel features in coordination chemistry. Some examples are; a whole category of oxygen based metallohelicates,^4–7^ an entire family of molecular platforms for the construction of supramolecular edifices,^3,8^ or a novel type of paddle wheel complexes.^9^ One subclass of this kind of ligands exhibits two β-diketone groups separated by an *m*-pyridinediyl spacer (Scheme 1A). Their interesting coordination chemistry is illustrated by an impressive family of heterometallic clusters with a chain-like [M–Ln–M]^7+^ core (M^2+^ = Cu, Ni; Ln^3+^ = any lanthanide) sandwiched by two ligands in the coordination mode shown in Scheme 1B.^10,11^

**Fig. 1:**
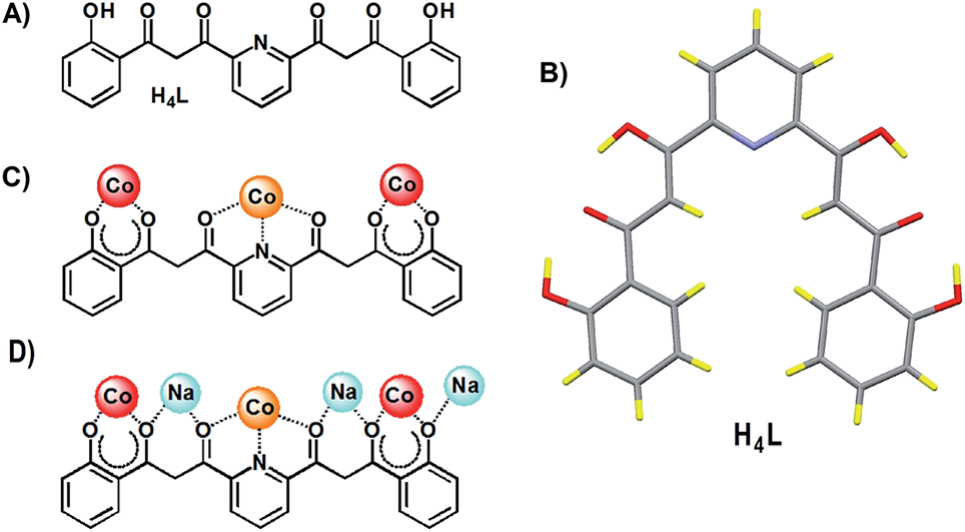
Bis-dicarbonyl form of ligand H_4_L (A), solid state molecular structure of H_4_L (C, grey; O, red; N, purple; H, yellow) showing its fully enolic form (B), and coordination modes featured by H_4_L in compounds **1** (C) and **2** (D).

We present here the unexpected (some unprecedented) features resulting from aerobic reactions of the related ligand 2,6-bis-(3-oxo-3-(2-hydroxyphenyl)-propionyl)-pyridine, H_4_L (Fig. 1A), with Co(ii) in pyridine, under basic conditions. This ligand had only been used once in the past, also with Co(ii).^12^ On that occasion, the chemistry was performed in the absence of any base, and the result was the formation of a cluster with formula [Co_8_O(OH)(H_2_L)_6_]NO_3_, which encapsulates a [μ_3_-O···H···μ_3_-O] moiety while the ligand H_4_L was found to retain its phenolic protons upon coordination. We show now that the use of strong basic conditions leads to full deprotonation of H_4_L, which is conducive to the oxidation of some of the Co(ii) ions to Co(iii) by atmospheric oxygen. This is likely the consequence of engaging the phenolate groups into coordination, thus stabilizing the latter ions. The combinations of reagents NBu_4_OH/Co(NO_3_)_2_ and NaH/Co(BF_4_)_2_, respectively, with H_4_L in pyridine have yielded the new clusters [Co_4_(L)_2_(OH)(py)_7_]NO_3_ (**1**) and [Co_8_Na_4_(L)_4_(OH)_2_(CO_3_)_2_(py)_10_](BF_4_)_2_ (**2**). The structural constrains resulting from this combination of metals and ligands have allowed to unveil quite remarkable features in coordination chemistry. One is a very rare example of a bridging “crevice” pyridine ligand (in complex **1**). The other consists of two carbonate ligand anions, forced to stay at an extraordinarily close distance to each other within cage **2**, to the point that the intermolecular O···O distance (1.946 Å) is found to be within 0.03 Å from the longest detected stable O–O bond (1.915 Å).^13^ These occurrences are studied in detail, through physical and theoretical methods.

## 2. Experimental

### 2.1 Synthesis

#### 2,6-Bis-(3-oxo-3-(2-hydroxyphenyl)-propionyl)-pyridine, H_4_L

This molecule was prepared as previously reported by our group.^14^ Crystals were obtained here by mixing H_4_L (20 mg) with CH_3_CN, CHCl_3_ or MeOH (4 mL) and heating to the boiling point of the solvent until complete dissolution and then letting the solution to slowly cool down. Crystals suitable for single crystal X-ray diffraction form after several minutes.

#### [Co_4_(L)_2_(OH)(py)_7_]NO_3_ (**1**)

A solution of H_4_L (50 mg, 0.12 mmol) and NBu_4_OH (0.6 mL of a 1 M methanolic solution, 0.6 mmol) in pyridine (15 mL) was added dropwise with continuous stirring to a solution of Co(NO_3_)_2_·6H_2_O (72.2 mg, 0.25 mmol) and Gd(NO_3_)_3_·6H_2_O (36.4 mg, 0.08 mmol) in pyridine (15 mL). The mixture was brought to reflux for 2.5 hours and then cooled down to room temperature. A brown solid was removed by filtration and the red solution was layered with ether (ratio 1 : 1.5 vol.). After two weeks, dark red crystals were collected and washed with ether and water to remove traces of the remaining ligand and salts. Final yields in the 8–21% range were obtained. IR (KBr pellet): *ν*/cm^–1^ = 3419 m, 3072 m, 1652 w, 1598 s, 1566 s, 1530 s, 1505 s, 1452 s, 1384 s, 1317 s, 1256 m, 1230 m, 1207 s, 1150 s, 1121 m, 1067 m, 1033 m, 958 w, 864 w, 754 s, 699 s, 668 m, 650 m, 584 m, 545 w, 490 m. Anal. calc. (Found) for **1**·5.5H_2_O (–1py): C, 54.1 (53.7); H, 4.0 (3.6); N, 7.5 (7.3).

#### [Co_8_Na_4_(L)_4_(OH)_2_(CO_3_)_2_(py)_10_](BF_4_)_2_ (**2**)

Co(BF_4_)_2_·6H_2_O (84.3 mg, 0.25 mmol) was dissolved in pyridine (15 mL). An orange solution of H_4_L (50 mg, 0.12 mmol) and NaH (24.8 mg of mineral oil 60%, 0.63 mmol) in pyridine (15 mL) was added dropwise to the above solution while stirring. The dark orange solution was stirred at room temperature for 150 min. The resulting dark red solution was filtered and the orange filtrate was layered with ether (volume ratio 1 : 1.5). Dark red crystals appeared after two weeks and were separated by filtration and washed with ether and water to remove traces of the remaining ligand and salts. Final yields in the 11–18% range were obtained. IR (KBr pellet): *ν*/cm^–1^ = 3431 m, 3069 m, 1652 w, 1635 w, 1599 s, 1566 s, 1531 s, 1506 s, 1455 s, 1386 m, 1317 s, 1245 w, 1207 m, 1150 s, 1122 w, 1066 m, 1032 m, 957 m, 864 w, 754 s, 697 s, 668 m, 650 m, 584 m, 547 w, 489 m. Anal. calc. (Found) for **2**·3H_2_O·2py: C, 54.1 (53.9); H, 3.6 (3.7); N, 6.6 (7.0).

### 2.2 X-Ray crystallography

Data for ligand H_4_L and for compound **1** were collected, respectively, on a yellow needle and on a red block at 150 K on a Bruker APEX II CCD diffractometer on Advanced Light Source beamline 11.3.1 at Lawrence Berkeley National Laboratory, from a silicon 111 monochromator (*λ* = 0.7749 Å). Data were collected for compound **2** on an orange plate at 100 K on a Bruker APEX II QUAZAR diffractometer equipped with a microfocus multilayer monochromator with Mo Kα radiation (*λ* = 0.71073 Å). Data reduction and absorption corrections were performed with SAINT and SADABS,^15^ respectively. The structures were solved with SIR97^16^ (H_4_L) and SHELX-TL^15,17^ (**1** and **2**) and refined on *F* ^2^ with SHELX-TL suite.^15,17^ In **1**, one of the oxygens of the nitrate ion is disordered over two equivalent positions. The atoms of both this nitrate ion and one pyridine molecule sitting on the symmetry operation were refined with displacement parameters restraints. In **2** one of the sodium atoms is disordered over two positions with similar occupation, while one of the coordinated pyridines is disordered over two positions sharing the same nitrogen (N7). These as well as oxygens coordinated to the disordered sodium atom and a number of carbon atoms from phenyl groups of the ligands and of coordinated pyridines were refined with displacement parameters restraints, due to disorder. Three of the four lattice pyridines also required the use of rigid body restraints for their refinement to converge, in addition to displacement parameters restraints. The tetrafluoroborate ion was refined with both distance and displacement parameters restraints. At the end of the refinement, there remained a number of weak electron diffraction peaks that seemed to form two partial and highly disordered lattice pyridine molecules. Their refinement was unstable even with strong displacement parameters restraints and the corresponding space was thus analyzed and taken into account with SQUEEZE as implemented in the PLATON package.^18^ A total of 310 electrons per cell were recovered by SQUEEZE, mostly over two voids of 580 cubic angstrom each. These figures are reasonable for at least six additional diffuse pyridine molecules per cell, *i.e.* three per [Co_8_] formula unit. These have been included in the formula.

### 2.3 Physical Measurements

Variable-temperature magnetic susceptibility data were obtained with a Quantum Design MPMS5 SQUID magnetometer. Pascal's constants were used to estimate diamagnetic corrections to the molar paramagnetic susceptibility. The elemental analysis was performed with a Elemental Microanalizer (A5), model Flash 1112 at the Servei de Microanàlisi of CSIC, Barcelona, Spain. IR spectra were recorded as KBr pellet samples on a Nicolet AVATAR 330 FTIR spectrometer. Positive ion ESI TOF mass spectrometry experiments were performed on a LC/MSD-TOF (Agilent Technologies) at the Unitat d'Espectrometria de Masses de Caracterització Molecular (CCiT) of the University of Barcelona. The experimental parameters were: capillary voltage 4 kV, gas temperature 325 °C, nebulizing gas pressure 15 psi, drying gas flow 7.0 L min^–1^, and fragmentor voltage ranging from 175 to 300 V. The samples (μL) were introduced into the source by an HPLC system (Agilent 1100), using a mixture of H_2_O/MeCN (1/1) as eluent (200 μL min^–1^).

## 3. Results and discussion

### 3.1 Synthesis

As mentioned in the Introduction, in absence of a base, H_4_L was found to react with a Co(ii) salt leading to a cluster where the phenol groups of the ligand remain protonated and do not coordinate.^12^ It has now been found that the use of a strong enough base allows removing all the ionisable protons from H_4_L, which facilitates the involvement of the resulting phenolate groups in the coordination. This concept had been proofed previously with the related ligand H_4_L1, featuring an *m*-phenylene spacer instead of the *m*-pyridinediyl. In that case, the presence of AcO^–^ allowed only removing the β-diketone protons, leading to complexes with a [M_2_(H_2_L1)_2_] core.^19^ Instead, stronger bases such as NBu_4_OH or NaH react also with the phenols of H_4_L1, serving to engage more metals to the coordination with formation of linear molecules of the type [M_4_(L1)_2_].^20,21^ Here the reactivity becomes richer. Full deprotonation of H_4_L seems to favour the oxidation of some of the Co(ii) ions to Co(iii) with atmospheric oxygen (see structural analysis) by stabilization of the latter ions through chelation. Thus the reaction between H_4_L and Co(NO_3_)_2_ in pyridine, in the presence of NBu_4_OH, leads to the formation of a new cluster, [Co_4_(L)_2_(OH)(py)_7_]NO_3_ (**1**). It must be mentioned that the procedure was originally intended to incorporate a lanthanide ion together with cobalt, therefore it was conducted in the presence of Gd(NO_3_)_3_. However, the rare earth has never been observed in the isolated product. On the other hand, the absence of gadolinium salt prevents the formation of any crystals or identifiable products. It is however not clear what the precise role of this component is in the equilibrium. While the formation of **1** involves presumably other side reactions, it can be described with a net equation as originating from the starting materials (eqn (1)).

1 4Co(NO_3_)_2_·6H_2_O + 2H_4_L +
0.5O_2_ + 7NBu_4_OH + 7py →
[Co_4_(OH)(L)_2_(py)_7_](NO_3_) +
7NBu_4_NO_3_ + 31H_2_O

The use of NaH as a base in a very similar reaction entails profound differences to the product obtained. Thus, mixing NaH, H_4_L and Co(BF_4_)_2_ in pyridine allows crystallization of the assembly [Co_8_Na_4_(L)_4_(OH)_2_(CO_3_)_2_(py)_10_](BF_4_)_2_ (**2**), featuring the same Co(ii) to Co(iii) ratio as in complex **1**. In this case, the presence of Na(i) ions plays a key role resulting in the “dimerization” of the basic [Co_4_L_2_(OH)]^+^ unit already observed in the tetranuclear complex (see below). The reaction involves oxidation of Co(ii) by atmospheric oxygen and the capture of CO_2_ from air through conversion to CO_3_^2–^ or HCO_3_^–^. This process, favored by strong basic conditions and coordination to metals has been widely documented.^22–24^ In one of the few mechanistic studies performed,^25^ it is proposed that it occurs following the insertion of CO_2_ within the Ni–O coordination bond of a terminal hydroxide from a Ni(ii) square planar mononuclear complex. However, this reaction has been more commonly observed on precursors containing bridged M(ii)_2_(OH)_1,2_ moieties.^26,27^ This is likely to be also the case in complex **2** since it contains Co(ii)_2_(μ-OH) moieties (see below). Other schemes involving three metals seem to proceed first by a nucleophilic attack of bound OH to CO_2_, which subsequently coordinates to the other two metals, yielding a μ_3_-CO_3_^2–^ ligand.^28^ The chemical process leading to complex **2**, starting from the initial reagents, can be described with a balanced equation (eqn (2)).

2 8Co(BF_4_)_2_·6H_2_O + 4H_4_L +
O_2_ + 2CO_2_ + 18NaH + 10py →
[Co_8_Na_4_(OH)_2_(L)_4_(CO_3_)_2_(py)_10_](BF_4_)_2_
+ 14NaBF_4_ + 18H_2_ + 46H_2_O

In both reactions, the yields of isolated crystals are relatively low. Thus, eqn (1) and (2) are only means of describing the possible processes of formation of **1** and **2**, respectively, without implying that other processes and equilibria are not also occurring. The main focus here is analyzing and describing the fascinating novel coordination features unveiled within these new compounds. Once isolated, the crystals could be re-dissolved in various solvents (acetone, MeOH, ACN, DMF). The nature of the systems in acetone solution was analyzed by positive ion mass spectrometry (Fig. S1–S4†). While the whole cluster cation was not observed for any of the compounds, in both cases it was possible to identify numerous forms of the [L_2_Co_4_] basic unit bearing H_2_O and/or pyridine ligands and also exhibiting several distributions of +2 and +3 oxidation states of the Co centers (*e.g.* [L_2_Co_4_]^2+^, [L_2_Co_4_(py)_2_]^2+^, [L_2_Co_4_(py)_2_]^2+^ [L_2_Co_4_(py)(H_2_O)_2_]^2+^, *etc.*). Some fragments lacking one of the central metal ions were also observed (such as [L_2_Co_3_] + 2H^+^, [L_2_Co_3_] + H^+^, [L_2_Co_3_(py)] + 2H^+^) as well as moieties incorporating a K^+^ into that vacant position ([L_2_Co_3_K(py)]^+^ + H^+^, [L_2_Co_3_K(py)(MeCN)]^+^ + H^+^, [L_2_Co_3_K(py)] + H^+^, [L_2_Co_3_K(py)(MeCN)]^+^, [L_2_Co_3_K(py)_3_(H_2_O)_2_]^+^, *etc.*, the presence of K^+^ and MeCN being inherent to the technique and thus very common). From this point of view, complex **2** in solution is essentially no different than compound **1**. These results indicate that a prevalent moiety in solution is most likely a solvated form of the [L_2_Co_4_]^2+^ rhombic fragment.

### 3.2 Description of structures

#### H_4_L

**Fig. 2:**
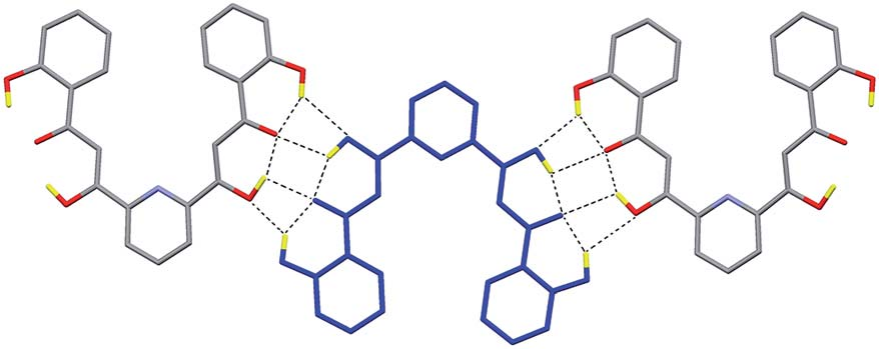
Representation of three molecules of H_4_L emphasizing the various three-center hydrogen bonding interactions established between them.

The solid state molecular structure of H_4_L has now been determined by single crystal X-ray diffraction (Table S1†), which shows that in the crystal, the molecule is fully in an enolic form (Fig. 1B and S5†), as was previously observed in a chloroform solution using ^1^H NMR.^14^ This tautomer is perhaps favored by a series of complementary three-center hydrogen bonds (Fig. 2), which add to the numerous π···π contacts established between the molecules in the crystal (Fig. S6†). The intra- and intermolecular bonding parameters of this structure are listed in Tables S2 and S3.†

#### [Co_4_(L)_2_(OH)(py)_7_]NO_3_ (**1**)

**Fig. 3:**
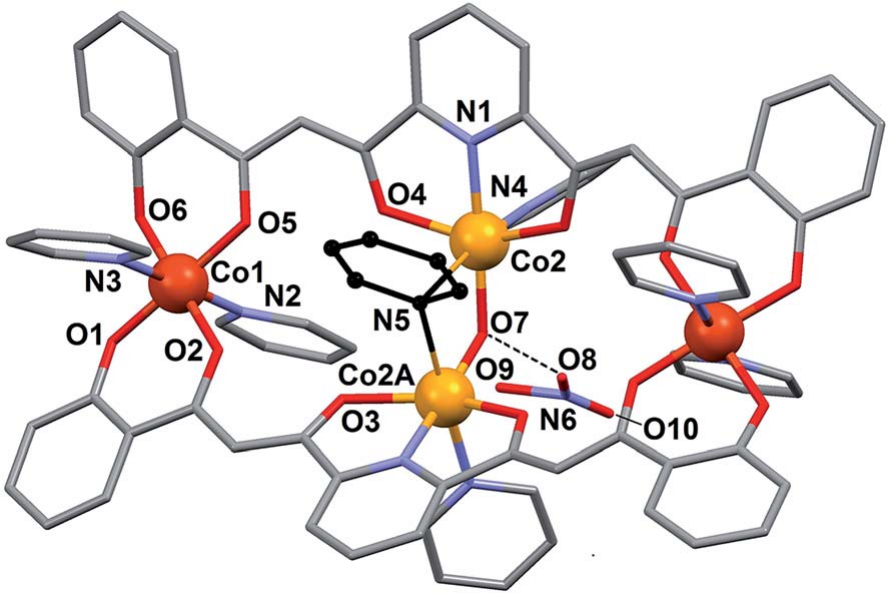
Molecular structure of [Co_4_(L)_2_(OH)(py)_7_]NO_3_ (**1**) with crystallographically unique heteroatoms labelled. The carbon atoms are in grey except these of the central μ-pyridine group, which have been emphasized in black. The hydrogen atoms are not shown. Only one of two disordered positions of NO_3_^–^ and μ-pyridine are shown.

Compound **1** crystallizes in the *C*2/*c* space group (Table S1†). Its structure consists of one cluster cation with charge +1 together with one nitrate group (Fig. 3). The metric parameters of this complex are listed in Table S4.† The asymmetric unit is formed by one half of the formula content and three molecules of pyridine, whereas the unit cell includes eight such units. The complex cation [Co_4_(OH)(L)_2_(py)_7_]^+^ is formed by two Co(iii) and two Co(ii) ions describing a very anisotropic rhombus. The long diagonal links the trivalent metal ions, and is spanned by two μ_3_-L^4–^ ligands that lie opposite each other and chelate both metals through their external ketophenolate moieties. Each of these ligands coordinates, through the central dipicolinate-like ONO pocket, to one of both Co(ii) metals defining the short diagonal, which is spanned by one μ-OH^–^ group and a remarkable bridging pyridine ligand (μ-py). The octahedral geometry of each Co(iii) center (Co1 and symmetry equivalent, s.e.) is completed by two axial pyridine ligands, lying *trans* to each other, while the very distorted octahedron of coordination around the Co(ii) ions (Co2 and s.e.) comes about with the concurrence of one terminal pyridine group per metal, lying in *trans* to the bonds with the μ-py group.

**Fig. 4:**
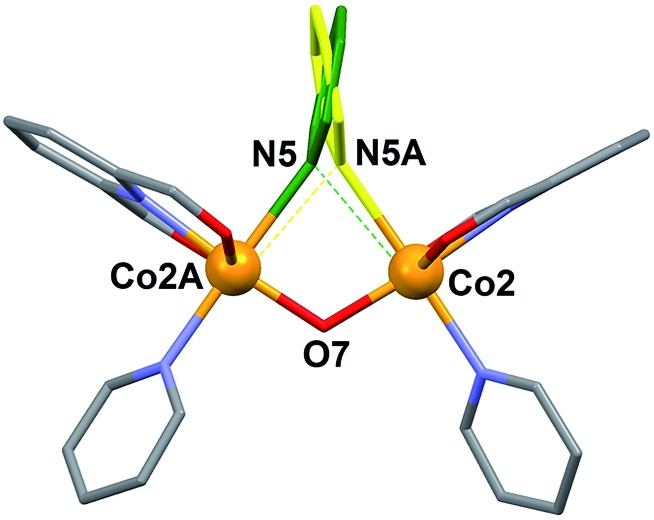
Representation of the central core of [Co_4_(L)_2_(OH)(py)_7_]NO_3_ (**1**) emphasizing the two positions of the disordered μ-pyridine group (yellow and green).

The NO_3_^–^ counter ion is disordered, pivoting around the N atom over two slightly different orientations and forming a hydrogen bond with the μ-OH^–^ ligand. The oxidation states postulated for the Co(ii) ions are consistent with the charge of the cluster and were very clearly confirmed by bond valence sum (BVS) analysis (Table S5†). Of all the unusual structural features of compound **1**, perhaps the most remarkable is the presence of a bridging pyridine ligand in between two Co(ii) centers (see the details in Table 1). This bridge interacts with both Co(ii) ions in a slightly asymmetric manner, thus featuring a shorter (2.367(5) Å) and a longer (2.700(5) Å) Co–N distance. In fact the occupation of this pyridine group within the crystal lattice is shared in equal amounts over two symmetric orientations corresponding to having the N donor closer to either one or the other Co(ii) ion (Fig. 4). These two orientations form a mutual calculated angle of 20.18°. In addition, the angles of each ring with the idealized equatorial planes around the Co(ii) ions are 45.48° and 65.65°, respectively. The molecule exhibits a crystallographic *C*_2_ axis passing through the donor atoms of the μ-OH ligand and bisecting the two orientations of the disordered μ-py group.

**Table 1:** Distance (Å) and angles (°) describing the bridging pyridine moiety in the structure of **1**, together with parameters derived from DFT calculations (see text). The binding energies are in kcal mol^–1^ ^*a*^

Co2–N5	2.367(4)	Co2–N5A–Co2A	80.32(10)
Co2–N5A	2.700(5)	Co2–O7–Co2A	116.22(11)
Co2–O7	1.9300(12)	Co–N calc.	2.214/2.861
Co2···Co2A	3.2774(7)	Binding energy	–38.8/–33.6

^*a*^Symmetry operation A: 1 – *x*, *y*, 0.5 – *z*.

This peculiar bridging interaction of pyridine with two metals has been termed a “crevice” interaction and is extremely rare in the literature. It originates at the exposed two-site “cleft” of a molecular scaffold in the absence of any better bridging ligand. It was observed for the first time on a dinuclear Mo(v) complex,^29,30^ and since then, very few further examples have been reported involving Ag(i),^31^ Ti(iv),^32^ Cs(i),^33^ or Cu(i).^34^ Here we study it by means of theoretical methods for the first time (see below).

#### [Co_8_Na_4_(L)_4_(OH)_2_(CO_3_)_2_(py)_10_](BF_4_)_2_ (**2**)

**Fig. 5:**
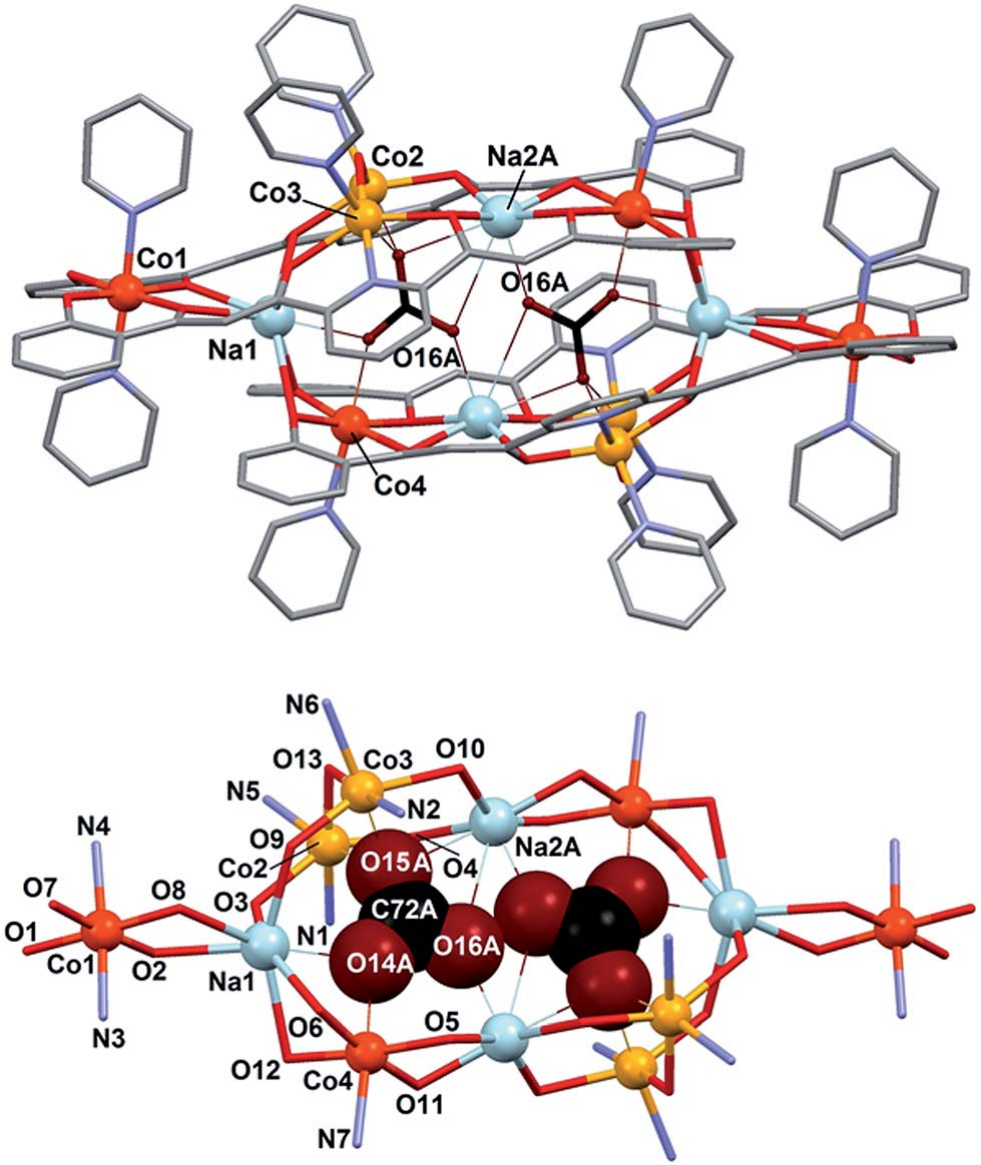
(top) Representation of the cation of [Co_8_Na_4_(L)_4_(OH)_2_(CO_3_)_2_(py)_10_](BF_4_)_2_ (**2**), with unique metals and closest O atoms from CO_2_^3–^ labelled. Color code: grey, C; red, O; purple, N; orange, Co(ii); dark orange, Co(iii); blue, Na; CO_3_^2–^ emphasized in dark red and black. The hydrogen atoms are not shown. Only one position of the disordered species is shown. (bottom) Core of complex **2** with unique atoms labelled. The closest positions of the encapsulated CO_3_^2–^ ions, of the two disordered locations resolved are shown and emphasized in a space filling format.

This complex crystallizes in the space group *P*2_1_/*c* (Table S1†). The asymmetric unit contains one half of the formula unit (the latter including also ten pyridine molecules of crystallization), whereas the unit cell includes two full molecules and the corresponding amount of pyridine solvate molecules. The main molecule is formed by a centrosymmetric [Co_8_Na_4_(L)_4_(OH)_2_(CO_3_)_2_(py)_10_]^2+^ complex cation and two BF_4_^–^ groups. The cluster (Fig. 5, Table S6† for metrics) comprises two rhombic tetranuclear [Co(ii)_2_Co(iii)_2_] units very similar to that featured in **1** (see above), each bound to three additional Na(i) ions; two of them *via* the β-diketonate groups of the L^4–^ ligands and the third one through the end phenolate oxygen atoms of these ligands (see in Fig. 1D, the coordination mode of L^4–^). Two of these ions are in fact shared by both [Co_4_] rhombuses thus acting as the link between them. BVS analysis (Table S7†) clearly indicates that Co1 is in the oxidation state +3, whereas Co2 and Co3 are +2. However, the sum for Co4 seems ambiguous as to whether it is +2 or +3. Possible reasons for bonds slightly longer than expected for Co(iii) are the strains related with the dimerization through the Na^+^ ions and longer bonds to carbonate (see below), or more significantly, the detrimental effect of employing atom positions from a disordered structure. In any case, charge balance and the magnetic properties (see below) are fully consistent with the postulated [Co(ii)_2_Co(iii)_2_] distribution of oxidation states.

In fact, the [Co_8_] cages are distributed over two equally populated and very similar disordered positions (Fig. S7†). The cage offers the conditions to encapsulate two CO_3_^2–^ anions, which are brought to lie extremely close to each other in both disordered positions (O16A···O16A# = 1.946 Å and O16B···O16B# = 1.971 Å, respectively) considering the sum of the van der Waals radii for oxygen (*r*_V(O)_ = 1.4 Å). Both CO_3_^2–^ groups are stabilized within the cage by interactions with the metals (see details in Table 2). In one of the disordered positions the number of interactions is six; three Na(i), two Co(ii) and one Co(iii) cations. In the other, the sodium atom Na2 is slightly removed away from the cage (distant by 0.912(8) Å from the first position, Fig. S7†), and thus loses contact with the internal CO_3_^2–^ ions. In comparison to cluster **1**, the coordination geometry of the Co(ii) ions (Co2 and Co3) is also distorted octahedral, replacing the μ-py group with a bridging oxygen atom from one CO_3_^2–^ ligand. Half of the Co(iii) centers have the same environment as in **1** (Co1), whereas the other half (Co4) replace one axial pyridine ligand by one oxygen atom from CO_3_^2–^ on that position. Encapsulation of CO_3_^2–^ from atmospheric carbon dioxide under strong basic conditions by incorporation into transition metal complexes is now very well documented.^35–37^ Fixation of more than one carbonate unit by one molecule is much rarer. In such cases, these species usually act essentially as spacers between metals or are subtended by metal ions conforming the external surface of a cage.^38–46^ In lesser occasions, the incorporated CO_3_^2–^ moieties may be rather considered as being encapsulated inside the coordination cage.^47–51^ In any case, two carbonate anions have never been forced to lie so close to each other as within complex **2**. To the best of our knowledge, the closest intermolecular contact between CO_3_^2–^ species observed to date (2.487 Å) was found within the compound [Y(H_2_O)]_2_(C_2_O_4_)(CO_3_)_2_, from a structure resolved by powder diffraction methods.^52^ The occurrence here is quite remarkable since the O···O contact now observed through single crystal X-ray diffraction methods is very close (within 0.03 Å) to the covalent O–O distance detected by spectroscopic methods on the molecule HOON, found to be stable at near 2 K. This distance was calculated to be, from the experimental data, of 1.915 Å.^13^ Therefore, this limiting observation and the one now reported represent the meeting point in the oxygen–oxygen distance when coming from two ends, that of covalent interactions and that of (forced) intermolecular contacts.

**Table 2:** Distance (Å) and angles (°) describing CO_3_^2–^ ions interactions with core metal ions in the structure of **2**, suffixes A and B correspond to the two disordered positions of the CO_3_^2–^ ions^*a*^

O14A–Na1#	2.230(18)	Co4–O14A–Na1#	93.7(6)
O14A–Co4	2.088(15)	Co3#–O15A–Co2#	99.0(5)
O15A–Co3#	1.958(14)	Co3#–O15A–Na2A#	95.8(7)
O15A–Co2#	2.185(16)	Co2#–O15A–Na2A#	92.2(5)
O15A–Na2A#	2.785(19)	Na2A–O16A–Na2A	138.8(6)
O16A–Na2A	2.241(14)	Co4–O14B–Na1	90.6(6)
O16A–Na2A#	2.970(18)	Co2–O15B–Co3	91.6(4)
O14B–Na1#	2.320(18)	Co2–O15B–Na2B	85.9(4)
O14B–Co4	2.110(14)	Co3–O15B–Na2B	84.0(4)
O15B–Co2#	2.151(14)		
O15B–Co3#	2.248(10)	O16A···O16A#	1.946
O15B–Na2B#	2.803(14)	O16B···O16B#	1.971
O16B–Na2B	2.991(16)		

^*a*^Symmetry operation #: 1 – *x*, 1 – *y*, 1 – *z*.

### 3.3 DFT calculations

The extremely rare coordination interactions observed here warrant a proper description through a theoretical treatment. For this we employed density functional theory (DFT) calculations.^53^

The energy of the “crevice” pyridine has indeed not been studied theoretically yet. The original papers, reporting a Mo–(μ-py)–Mo moiety,^29,30^ speculate about the existence or not of a Mo···py interaction, in view of very long Mo–N distances (2.967 Å and 2.931 Å). When found bridging two Ag(i) ions,^31^ the pyridine group was described as “weakly coordinating”, with Ag–N of 2.71 Å. The complex involving Ti(iv),^32^ is the only reported example where the bridging pyridine has been crystallographically solved as disordered over two equivalent positions, showing two distinctly different (2.532 Å and 3.093 Å) Ti–N distances, as found here in complex **1**. In fact, the original solution for the structure of **1** featured a symmetric μ-pyridine ligand. It was in light of the simulation procedure (see below) that the data were refined anew and the disorder unveiled. Thus, the nuclear configuration of [Co_4_(OH)(L)_2_(py)_7_]^+^ was optimized by means of DFT calculations carried out with Gaussian 09 (ref. 54) using the B3LYP^55^ functional within the spin unrestricted formalism, together with an Ahlrichs SVP basis set^56^ on all atoms and Grimme's D2 empirical dispersion correction.^57^ The result of this optimization showed the μ-py group in a very asymmetric configuration, with very differentiated N–Co distances (2.214 Å and 2.861 Å) and two distinct orientations of the ring with respect to the Co(ii) equatorial planes (85.70° and 18.67°). This observation prompted the new refinement of the experimental crystallographic data (see above), which unveiled that this group is indeed bound unsymmetrically (Fig. 4), although not so much as suggested by the simulation. These differences could be explained to a large extent by packing effects. DFT binding energies were then computed at the B3LYP-D2/TZVP level. In these calculations, the basis set superposition error was corrected using the counterpoise method.^58^ A binding energy of –38.6 kcal mol^–1^ was first determined for the μ-py group by using as a model a truncated version of the optimized structure (Fig. S8†), chosen to avoid the inclusion of the distal metals, not relevant for this calculation, since they are too distant to have an influence on the binding energy of interest. Subsequently, calculations were performed employing the same simplified model, using now the coordinates of the experimental solid state structure for all the nuclear positions, except these from μ-py. A very similar value of –33.6 kcal mol^–1^ was reached. For comparison, the binding energy of terminal pyridine to the distal Co(iii) ion was determined by DFT calculations on a fragment of **1** containing the relevant metal (Fig. S8†) and using the experimental coordinates of the atoms involved (with Co–N of 1.943 Å). The calculated value is –41.0 kcal mol^–1^. This means that the binding energy of the μ-py group in **1** is comparable to that of a true terminal py ligand. The contribution of the individual metal–ligand interactions has been analyzed by calculating the critical points around the Co ions involved in this interaction (Co2 and Co2A) using the AIM method,^59^ using the experimental coordinates of one of the disordered components of the structure (Fig. S9†). A list of the critical points encountered and the electron density at these points is in Table S8.† It has been found that indeed there is a critical point for both Co–N vectors featured by the μ-py ligand, which shows that the ligand interacts with each of the metals. The electron density at these critical points is 3.75 × 10^–2^ and 1.75 × 10^–2^ a.u. for the short and long interaction, respectively. Since the electron density at the bond critical points correlates with the strength of the bond,^59^ 68% of the interaction energy (–22.8 kcal mol^–1^) can be attributed to the short contact and 32% (–10.8 kcal mol^–1^) to the long one.

**Fig. 6:**
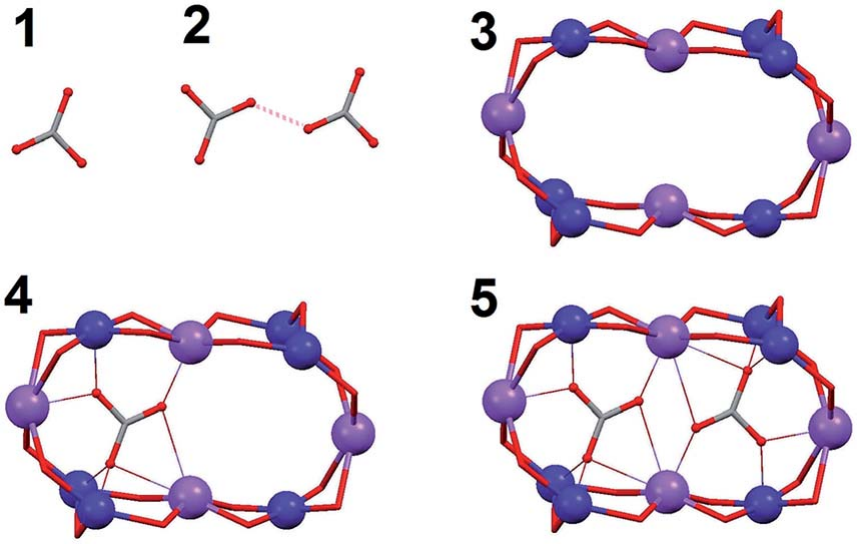
Simplified scheme of the models used for DFT calculations: ‘1’, a free CO_3_^2–^ anion (*E*_1_); ‘2’, a dimer of two CO_3_^2–^ anions (*E*_2_); ‘3’, the full cluster anion of **2** without the CO_3_^2–^ ligands (*E*_3_); ‘4’, the entire cluster anion of **2** with only one CO_3_^2–^ ligand (*E*_4_); ‘5’, the cluster of **2** with both encapsulated CO_3_^2–^ groups (*E*_5_). All species have been calculated in the gas phase and their energies obtained at the B3LYP-D2/SVP level.

The cluster cation of **2** exhibits the shortest non-covalent O···O distance ever observed between two CO_3_^2–^ species. The reason that these two species come so close to each other is the stabilization brought by the large number of interactions that they establish with the metals of **2** upon coordination. DFT calculations constitute an invaluable tool to verify and quantify this hypothesis. Thus, the absolute energy of various model systems (Fig. 6) built up using the experimental coordinates of the pertinent atoms of **2** was determined. For this, the atomic positions of the component that locates the CO_3_^2–^ anions closest to each other (distance O···O, 1.946 Å) was employed (Fig. 5). The energies associated to the other components were not expected to vary significantly (see below). First, the energy of bringing two CO_3_^2–^ anions at the distance observed within **2** in the same relative orientation (*E*_dimer/out_ = *E*_2_ – 2*E*_1_; Fig. 6 and S10†) without considering any other interaction, is extremely high; +349.6 kcal mol^–1^. This renders as quite remarkable the observation of these two anions in such relative positions within the cage. The stabilization attained upon coordination of CO_3_^2–^ inside the cage was estimated by calculating the energy of encapsulating one such anion from the gas phase into the cluster (*E*_coord1_ = *E*_4_ – *E*_3_ – *E*_1_; Fig. 6 and S10†), which amounts to –773.4 kcal mol^–1^. This already suggests that the system is to release energy when including two CO_3_^2–^ inside that cavity, despite the cost of having them so close to each other. Likewise, bringing two infinitely distant carbonate molecules inside the cage (*E*_coord2_ = *E*_5_ – *E*_3_ – 2*E*_1_; Fig. 6 and S10†) also represents an important gain in stability, the energy of the process being calculated as –1329.4 kcal mol^–1^, consistent with the experimental observation. The process as calculated is not perfectly comparable with the real situation, since the species involved are not in the gas phase but in pyridine solution. Nevertheless, a medium made of pyridine molecules, which are good Lewis bases, should favor the encapsulation even further. The models studied also allow to quantify the repulsion of the CO_3_^2–^ groups once they are inside the cage (*E*_dimer/in_ = *E*_coord2_ – 2*E*_coord1_ = *E*_5_ + *E*_3_ – 2*E*_4_; Fig. 6 and S10†). Thus the interaction involves an energy of +217.5 kcal mol^–1^. While this unfavorable interaction remains relatively high, it is reduced by 38% as compared to the cost of maintaining two CO_3_^2–^ ions at such distance in the gas phase. This is because the interactions with the metals withdraw an important part of the negative charge from the anions, diminishing the magnitude of their mutual repulsion when they are inside the cage. This last calculated value does not depend on the medium outside the cage, since the models used never involve free CO_3_^2–^. The conclusions arising from these calculations are not expected to vary at all if the atomic coordinates of other disordered components present in the crystal lattice (Fig. S7†) were employed. To illustrate this, *E*_dimer/out_ was calculated using the positions of CO_3_^2–^ in this other component and a value of +365.6 kcal mol^–1^ was extracted, only 16% higher than for the component chosen to illustrate the interaction energies in **2**.

### 3.4 Bulk magnetization properties

**Fig. 7:**
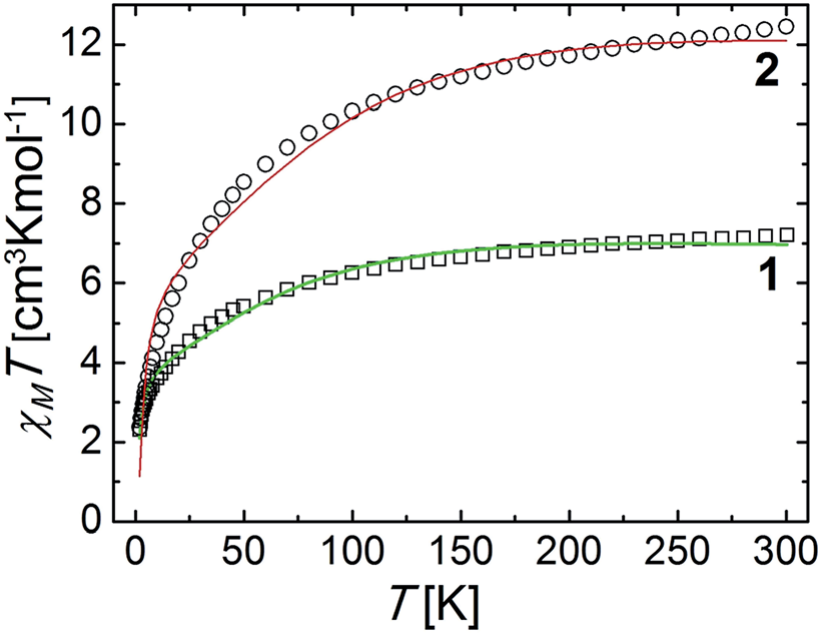
Plots of *χ*_M_ *T vs. T* for complexes **1** and **2**. The solid lines are best fits to the experimental data (see text for details).

Complexes **1** and **2** exhibit one and two [L_2_Co_4_] moieties in their molecule, respectively. The metals in these units are distributed in the form of a rhombus (Fig. 3) with two Co(iii) ions (Co1 and symmetry equivalent) spanning the long diagonals and two Co(ii) centres (Co2 and Co2A) at the ends of the short one. The trivalent metals are expected to be diamagnetic (*S* = 0) whereas the Co(ii) centres, bridged by one μ-OH^–^ and the μ-py ligand (or one O-atom from CO_3_^2–^), must be paramagnetic. Variable temperature magnetization measurements were performed on powdered microcrystalline samples of both compounds under a constant magnetic field of 0.5 T. The results are shown in Fig. 7, in the form of *χ*_M_ *T vs. T* plots (*χ*_M_ is the molar paramagnetic susceptibility). At 300 K, the *χ*_M_ *T* product values are 7.21 and 12.45 cm^3^ Kmol^–1^, respectively, much higher than those expected for two and four uncoupled high spin (*S* = 3/2) Co(ii) centers (expected at 3.75 and 7.5 cm^3^ Kmol^–1^, respectively, for *g* = 2). This means that the magnetic properties are strongly affected by the orbital angular momentum of these ions, not quenched despite the significant deviation from the octahedral geometry shown by them. *χ*_M_ *T* decreases as the temperature declines, increasingly faster towards lower temperatures, to reach 2.30 and 2.37 cm^3^ Kmol^–1^, respectively, at 2 K. This may be due to the effects of spin orbit coupling, but also to a possible interaction between the two Co(ii) ions within each rhombus (Co2 and Co2A for **1** and Co2 and Co3 for **2**). The magnetic data were fit by matrix diagonalization of the Hamiltonian in eqn (3), using the program PHI.^60^

3 *Ĥ* = 2*λσL*_Co_*ŝ*_Co_ – 2*J*(*ŝ*_Co1_*ŝ*_Co2_) + 2*μ*_B_(*σL*_Co_ + *g*_Co_*ŝ*_Co_)*B*

In this Hamiltonian, *L*_Co_ and *ŝ*_Co_ are, respectively, the orbital and spin angular momenta of Co(ii) (subscripts 1 and 2 refer to each of the two metals), while *g*_Co_ is the isotropic gyromagnetic ratio for this ion. The parameters *J*, *λ* and *σ* correspond, respectively, to the exchange-coupling constant between both metals, the spin–orbit coupling constant of Co(ii) and a combined orbital reduction parameter of this metal,^61^ whereas *μ*_B_ and *B* have the usual meanings. Reasonable fits (Fig. 7, solid lines) were obtained for the following parameters (in the **1**/**2** format); *J* = –0.40/–0.89 cm^–1^, *g* = 2.31/2.09 with fixed parameters of *λ* = –140/–180 cm^–1^ and *σ* = –1.0/–1.0. The discrepancies with the experimental data appear more noticeable in the temperature range between 35 and 100 K. This may be due to the approximations inherent to the model employed. In fact, treating the exchange between orbitally non-degenerate ions is very difficult. The approach used here considers only the coupling between true spin states, and not these of the orbital angular momentum.^62^ This is probably the reason why there are not magnetostructural correlations of exchanged coupled Co(ii) ions in the literature. Nevertheless, weak couplings are generally observed between such ions when linked by oxygen monoatomic bridges.^63^

## 4. Conclusions

By employing strong basic conditions in reactions of the ligand 2,6-bis-(3-oxo-3-(2-hydroxyphenyl)-propionyl)-pyridine, H_4_L, with Co(ii) salts, two mixed-valence Co(ii)/Co(iii) clusters have been obtained with unprecedented structures. The unconventional disposition of metals within these clusters prompts the isolation of one bridging, very rare “crevice” pyridine group in **1**. DFT calculations reveal a binding energy to each Co(ii) of approximately 40% of a regular Co–py coordination bond. The cage of **2** is seen to trap two CO_3_^2–^ anions that are held at the closest intermolecular distance ever seen for such species. It can be seen through calculations that the repulsion energy between these is strongly reduced inside the cage, by interaction with several Lewis acids, and that the system is very stable, thus rationalizing its formation. The very close lying CO_3_^2–^ groups inside **2** seem poised to easy oxidation and subsequent transformation into peroxodicarbonate. This suggests a possibility for catalytic CO_2_ capture from the atmosphere to form a reactive species, C_2_O_6_^2–^, useful for chemical synthesis.
