# Supplementary material for: New coordination features; a bridging pyridine and the forced shortest non-covalent distance between two CO3 2– species
Source: Chem Sci. 2014 Oct 8;6(1):123–31. doi: 10.1039/c4sc02491e (PMC5461881; doi:10.1039/c4sc02491e)
Supplement: Supplementary file 1 [file SC-006-C4SC02491E-s001.pdf]

Electronic Supplementary Material (ESI) for Chemical Science.
This journal is © The Royal Society of Chemistry 2014

Supporting Information

New coordination features; a bridging pyridine and the forced shortest non-covalent distance between two CO_3^{2-} species

V. Velasco,[a] D. Aguilà,[a] L. A. Barrios,[a] I. Borilovic,[a] O. Roubeau,[b] J. Ribas-Ariño,[c] M. Fumanal[c] and G. Aromí[a,]*

Positive Ion MS-ESI

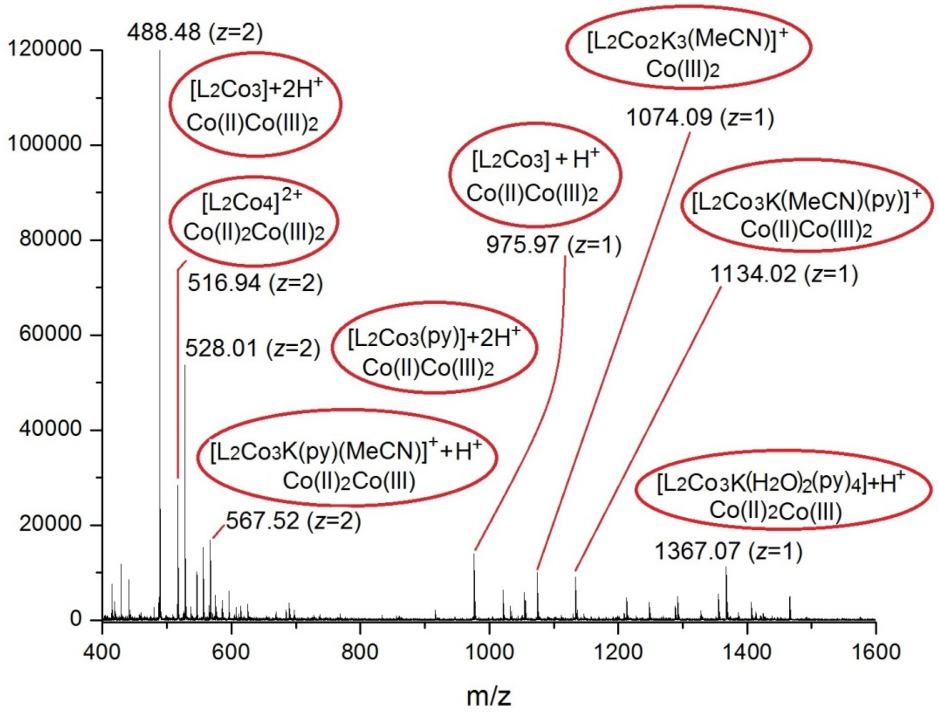

Figure S1. Positive Ion MS-ESI of $[Co_4(L)_2(OH)(py)_7]NO_3$ (**1**) in the 400-1600 range of *m/z*. The voltage employed was 215 V.

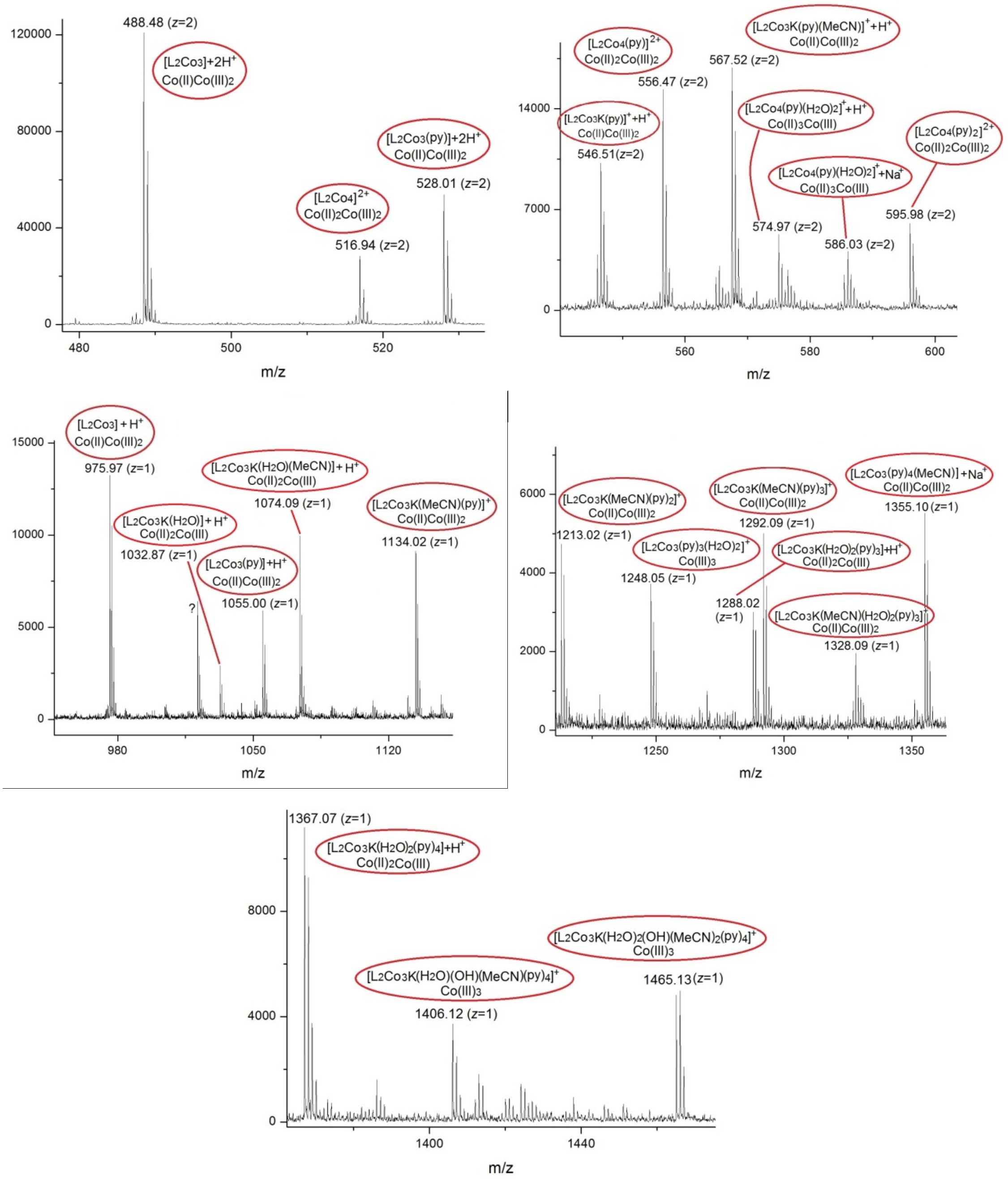

Figure S2. Positive Ion MS-ESI of $[Co_4(L)_2(OH)(py)_7]NO_3$ (**1**) 3mphasizing several ranges of *m/z*. The voltage employed was 215 V.

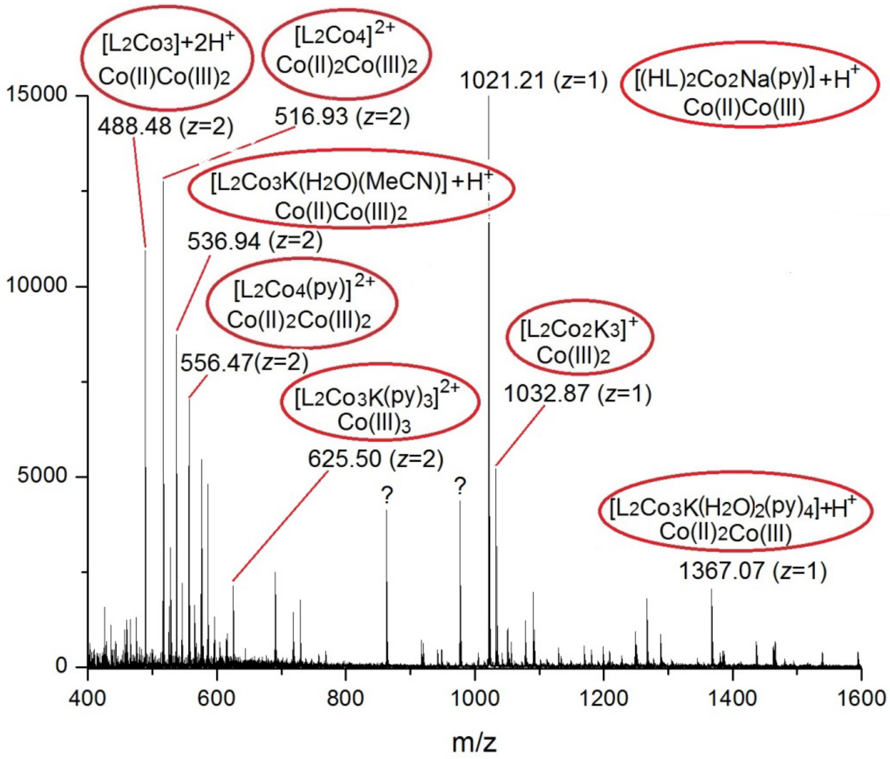

Figure S3. Positive Ion MS-ESI of $[Co_8Na_4(L)_4(OH)_2(CO_3)_2(py)_{10}](BF_4)_2$ (**2**) in the 400-1600 range of *m/z*. The voltage employed was 215 V.

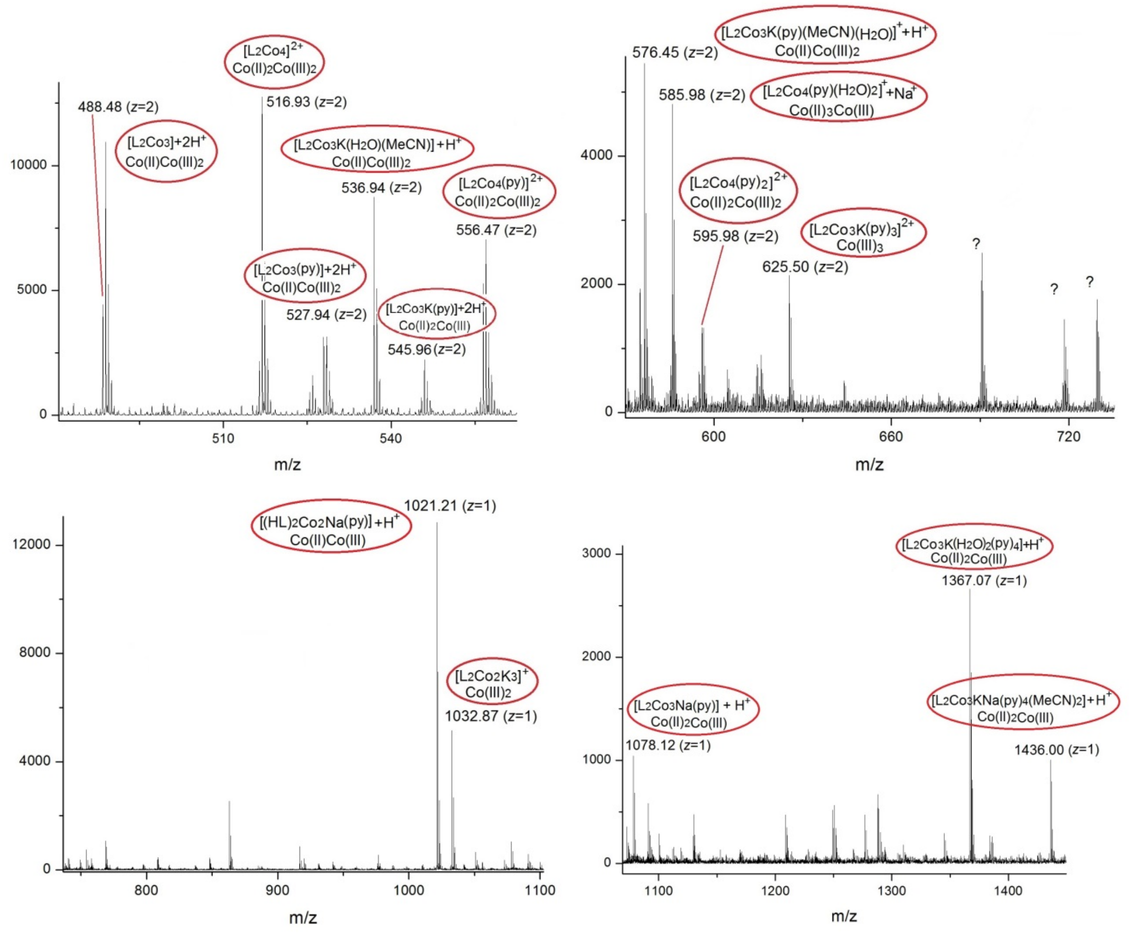

Figure S4. Positive Ion MS-ESI of $[Co_8Na_4(L)_4(OH)_2(CO_3)_2(py)_{10}](BF_4)_2$ (**2**) emphasizing several ranges of *m/z*. The voltage employed was 215 V.

Table S1: Crystal data and structure refinement for compounds **1** and **2**.

	H_4L	**1**·6py	**2**·10py
Formula	$C_{23}H_{17}NO_6$	$C_{111}H_{91}N_{16}O_{16}Co_4$	$C_{194}H_{158}N_{24}O_{34}B_2F_8Na_4Co_8$
M_r	403.38	2140.72	4106.47
Crystal system	Orthorhombic	Monoclinic	Monoclinic
Space group	Pbca	C2/c	$P2_1/c$
a (Å)	21.073(6)	16.833(2)	18.4101(7)
b (Å)	7.448(2)	19.112(2)	17.6277(7)
c (Å)	23.393(6)	30.840(4)	30.4003(12)
α (°)	90	90	90
β (°)	90	101.398(1)	106.776(2)
γ (°)	90	90	90
V (Å^3)	3671.6(17)	9726(2)	9445.9(6)
Z	8	4	2
ρ_{calc} (g/cm^3)	1.459	1.462	1.443
μ (cm^{-1})	0.128	0.940	0.776
colour/shape	Colourless/needle	Red/block	Orange/plate
Crystal size (mm^3)	0.11×0.02×0.01	0.35×0.25×0.20	0.56×0.37×0.08
λ (Å)	0.77490	0.77490	0.71073
T (K)	100	150	100
Reflections	2239	13526	17983
Unique reflections	1551	11941	13864
Parameters	283	688	1200
Restraints	0	32	504
R_1 (all data)^a	0.0782	0.0594	0.1037
R_1 [$I>2\sigma(I)$]^a	0.0454	0.0555	0.0885
wR_2 (all data)^b	0.1156	0.1798	0.2736
wR_2 [$I>2\sigma(I)$]^b	0.1009	0.1755	0.2614
S (all data)	0.992	1.090	1.059
S [$I>2\sigma(I)$]	0.992	1.084	1.030
Largest residuals (e/Å^3)	0.171/–0.252	1.381/–0.653	1.298/–1.541

^{a} $R_1 = \Sigma||F_o| - |F_c||/\Sigma|F_o|$. ^{b} $wR_2 = (\Sigma[w(F_o^2 - F_c^2)^2]/\Sigma[w(F_o^2)^2])^{1/2}$

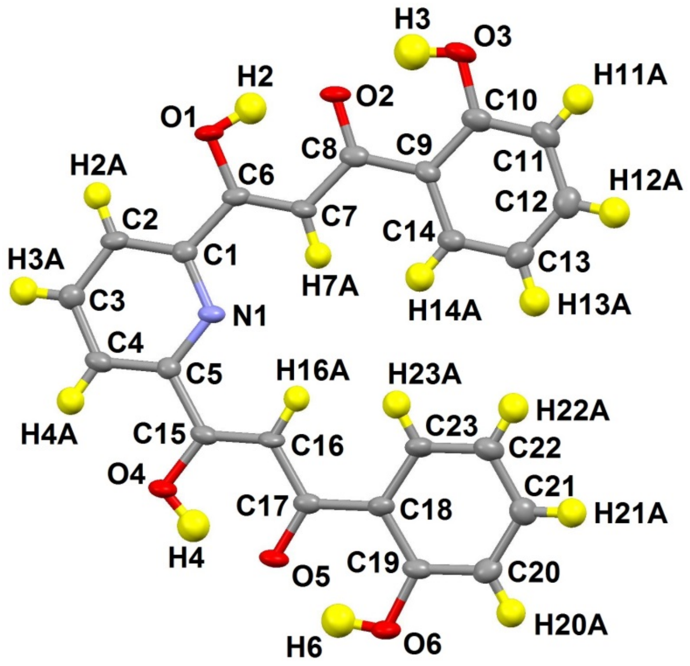

Figure S5. Labelled molecular representation of 2,6–bis–(3–oxo–3–(2–hydroxyphenyl)–propionyl)–pyridine, H_4L, with ellipsoids shown at the 50% probability level.

Table S2. Bond distances [Å] within ligand H_4L

O1–C6	1.347(4)	C8–C9	1.460(4)
O2–C8	1.269(4)	C9–C10	1.410(4)
O3–C10	1.356(4)	C9–C14	1.422(4)
O4–C15	1.339(4)	C10–C11	1.381(5)
O5–C17	1.274(4)	C11–C12	1.383(5)
O6–C19	1.362(4)	C12–C13	1.385(5)
N1–C1	1.342(4)	C13–C14	1.367(5)
N1–C5	1.348(4)	C15–C16	1.341(5)
C1–C2	1.385(4)	C16–C17	1.433(4)
C1–C6	1.481(4)	C17–C18	1.462(4)
C2–C3	1.386(4)	C18–C19	1.413(4)
C3–C4	1.389(4)	C18–C23	1.409(4)
C4–C5	1.375(4)	C19–C20	1.387(5)
C5–C15	1.488(4)	C20–C21	1.375(5)
C6–C7	1.339(4)	C21–C22	1.395(5)
C7–C8	1.438(4)	C22–C23	1.366(5)

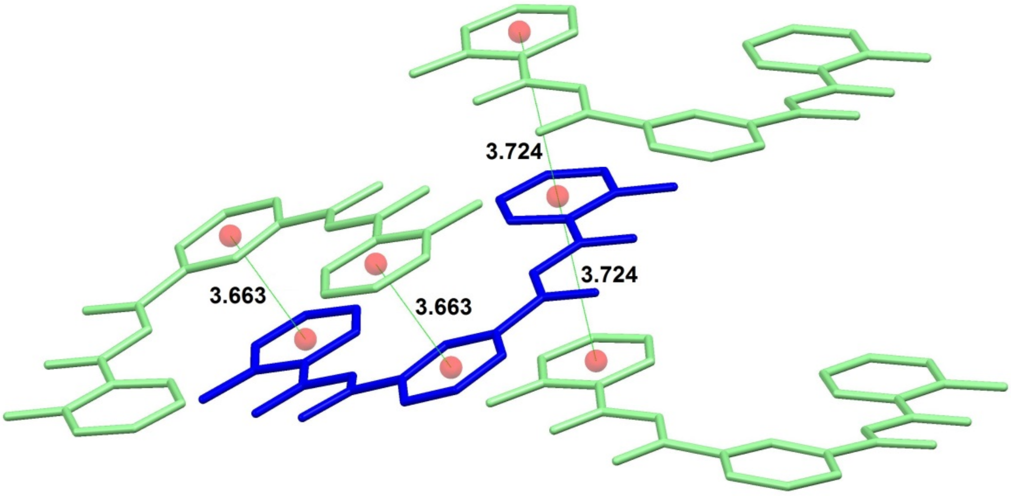

Figure S6. Representation of four molecules of H_4L emphasizing the four main π···π interactions that one molecule (here in blue) forms with its nearest neighbors (here in green). Centroid to centroid distances indicated. Hydrogen atoms not shown.

Table S3. Interatomic distances (Å) and angles (º) defining the hydrogen bonds in ligand H_4L.

O1···O2	2.587(3)	O1–H2···O2	157(3)
O1#···O5	2.871(3)	O1#–H2···O5	108(2)
O2···O3	2.580(3)	O3–H3···O2	149(3)
O2#···O4	2.825(3)	O4–H4···O2#	111(3)
O4···O5	2.581(3)	O4–H4···O5	151(3)
O5···O6	2.598(3)	O6–H6···O5	147(3)

Symmetry operation: #= ½+x, 1.5-y, 1-z

Table S4. Selected interatomic distances (Å) and angles (°) in the structure of $[Co_4(L)_2(OH)(py)_7]NO_3$ (**1**).

Co1–O1	1.8699(14)	O5–Co1–N3	91.52(7)
Co1–O6#	1.8733(15)	O2–Co1–N3	88.77(7)
Co1–O5#	1.8916(13)	O1–Co1–N2	91.16(7)
Co1–O2	1.8932(14)	O6–Co1–N2	91.99(7)
Co1–N3	1.9425(17)	O5–Co1–N2	87.41(7)
Co1–N2	1.9435(18)	O2–Co1–N2	87.00(7)
Co2–O7	1.9300(12)	N3–Co1–N2	175.67(7)
Co2–N1	2.0744(16)	O7–Co2–N1	175.70(7)
Co2–O3	2.0770(14)	O7–Co2–O3	104.96(4)
Co2–O4	2.0970(14)	N1–Co2–O3	75.27(6)
Co2–N4	2.1612(18)	O7–Co2–O4	103.92(4)
Co2–N5	2.367(4)	N1–Co2–O4	75.08(6)
Co2–N5A	2.700(5)	O3–Co2–O4	148.96(6)
		O7–Co2–N4	92.44(7)
Co1···Co2	6.1929(11)	N1–Co2–N4	91.81(7)
Co1···Co2A	6.2339(12)	O3–Co2–N4	95.68(6)
Co2···Co2A	3.2774(7)	O4–Co2–N4	94.23(6)
		O7–Co2–N5	86.17(11)
O1–Co1–O6	86.19(6)	N1–Co2–N5	89.57(11)
O1–Co1–O5	177.57(7)	O3–Co2–N5	85.8(11)
O6–Co1–O5	91.90(6)	O4–Co2–N5	85.0(2)
O1–Co1–O2	93.60(6)	N4–Co2–N5	178.22(15)
O6–Co1–O2	178.96(6)		
O5–Co1–O2	88.29(6)	Co2A–N5–Co2	80.32(10)
O1–Co1–N3	90.05(7)	Co2A–O7–Co2	116.22(11)
O6–Co1–N3	92.24(7)		

symmetry operation A: 1-x, y, -z+1/2

Bond Valence Sum (BVS) analysis

For each cation, BVS=$\sum_i e^{\left(r_0-r/_B\right)}$ over all the, *i*, bonds to the cation. The values of *B* and of r_0 for Co(II) and Co(III) bound to oxygen and nitrogen where taken from from iUCr data at http://www.iucr.org/resources/data/datasets/bond-valence-parameters, using file version 2013 (bvparm2013.cif). In red and green are the BVSs for Co(II) and Co(III), respectively.

Table S5. BVS analysis for $[Co_4(L)_2(OH)(py)_7]NO_3$ (**1**).

	r	r0 Co(II)	V Co(II)	r0 Co(III)	V Co(III)
Co1-O1	1.87	1.685	0.606531	1.637	0.53273576
Co1-O6	1.8733	1.685	0.601145	1.637	0.52800546
Co1-O5	1.8916	1.685	0.572136	1.637	0.50252589
Co1-O2	1.8932	1.685	0.569667	1.637	0.5003575
Co1-N3	1.9425	1.65	0.4536	1.75	0.59435989
Co1-N2	1.943	1.65	0.452987	1.75	0.59355724
BVSs			3.256066		3.25154175

	r	r0 Co(II)	V Co(II)	r0 Co(III)	V Co(III)
Co2-O7	1.93	1.685	0.515735	1.637	0.45298698
Co2-O3	2.077	1.685	0.346643	1.637	0.30446803
Co2-O4	2.097	1.685	0.328403	1.637	0.28844719
Co2-N1	2.0744	1.65	0.31758	1.75	0.41613034
Co2-N4	2.1612	1.65	0.251171	1.75	0.32911391
Co2-N5	2.367	1.65	0.144015	1.75	0.18870552
			1.903547		1.97985198

Table S6. Selected interatomic distances (Å) and angles (º) in the structure of $[Co_8Na_4(L)_4(OH)_2(CO_3)_2(py)_{10}](BF_4)_2$ (**2**).

Co1–O1	1.856(6)	Co1···Na1	3.528(2)	O4–Co2–O3	147.92(14)
Co1–O7	1.871(5)	Co2···Na1	3.888(2)	O13–Co2–O15B	79.3(3)
Co1–O8	1.889(4)	Co3···Na1	3.867(2)	O13–Co2–O15A	73.0(4)
Co1–O2	1.893(4)	Co4···Na1#	3.153(2)	N1–Co2–O15A	108.8(4)
Co1–N3	1.957(5)	Co2···Na2A	3.605(6)	N1–Co2–O15B	102.1(3)
Co1–N4	1.965(5)	Co3···Na2A	3.562(7)	O4–Co2–O15A	88.9(5)
Co2–O13	1.954(4)	Co4···Na2A	3.073(6)	O4–Co2–O15B	80.4(3)
Co2–N1	2.039(4)	Co2···Na2B	3.409(5)	O3–Co2–O15A	87.6(5)
Co2–O4	2.117(4)	Co3···Na2B	3.404(6)	O3–Co2–O15B	92.5(4)
Co2–O3	2.116(3)	Co4···Na2B	3.375(4)	O13–Co2–N5	91.45(17)
Co2–O15A	2.185(16)	Co1···Co2	6.2975(11)	N1–Co2–N5	86.94(18)
Co2–O15B	2.151(14)	Co1···Co3	6.2895(11)	O4–Co2–N5	98.21(17)
Co2–N5	2.178(5)	Co1···Co4#	6.1590(11)	O3–Co2–N5	93.69(16)
Co3–O13	1.949(4)	Co2···Co3	3.1537(10)	O15B–Co2–N5	170.1(17)
Co3–N2	2.044(4)	Co2···Co4	6.4594(10)	O15A–Co2–N5	164.0(4)
Co3–O10	2.138(4)	Co2···Co4#	5.6354(10)	O13–Co3–N2	177.45(19)
Co3–O9	2.137(4)	Co3···Co4	6.4719(10)	O13–Co3–O10	102.40(16)
Co3–O15A	2.158(14)	Co3···Co4#	5.5642(9)	N2–Co3–O10	75.19(16)
Co3–O15B	2.248(10)	Co1···Na1	3.528(2)	O13–Co3–O9	107.38(17)
Co3–N6	2.163(5)	Co2···Na1	3.888(2)	N2–Co3–O9	75.15(17)
Co4–O6	1.974(4)	Co3···Na1	3.867(2)	O10–Co3–O9	147.66(15)
Co4–O12	1.982(4)	Co4···Na1#	3.153(2)	O13–Co3–O15B	77.0(15)
Co4–O11	2.046(4)	Co2···Na2A	3.605(6)	O13–Co3–O15A	78.4(4)
Co4–O5	2.049(4)	Co3···Na2A	3.562(7)	N2–Co3–O15B	103.3(4)
Co4–N7	2.104(5)	Co4···Na2A	3.073(6)	N2–Co3–O15A	102.1(4)
Co4–O14A	2.088(15)	Co2···Na2B	3.409(5)	O10–Co3–O15B	81.6(4)
Co4–O14B	2.110(14)	Co3···Na2B	3.404(6)	O10–Co3–O15A	86.8(6)
		Co4···Na2B	3.375(4)	O9–Co3–O15B	92.9 (4)
Na1–O14A	2.231(18)	Co1···Co2	6.2975(11)	O9–Co3–O15A	87.0(5)
Na1–O14B	2.320(18)	Co1···Co3	6.2895(11)	O13–Co3–N6	89.50(17)
Na1–O9	2.370(4)	Co1···Co4#	6.1590(11)	N2–Co3–N6	90.09(18)
Na1–O3	2.408(4)	Co2···Co3	3.1537(10)	O10–Co3–N6	99.80(18)
Na1–O2	2.477(4)	Co2···Co4	6.4594(10)	O9–Co3–N6	92.87(17)
Na1–O12	2.496(5)	Co2···Co4#	5.6354(10)	O15B–Co3–N6	166.4(4)
Na1–O8	2.511(4)	Co3···Co4	6.4719(10)	O15A–Co3–N6	167.3(5)
Na1–O6	2.602(4)	Co3···Co4#	5.5642(9)	O6–Co4–O12	89.34(17)
Na2A–O16A	2.241(14)			O6–Co4–O11	172.47(16)
Na2A–O16B	2.102(16)	O1–Co1–O7	87.7(3)	O12–Co4–O11	90.19(15)
Na2A–O16A#	2.968(17)	O1–Co1–O8	177.90(19)	O6–Co4–O5	90.36(15)
Na2A–O16B#	3.073(19)	O7–Co1–O8	93.4(2)	O12–Co4–O5	174.44(16)
Na2A–O11	2.203(7)	O1–Co1–O2	93.9(2)	O11–Co4–O5	89.38(14)
Na2A–O5	2.271(6)	O7–Co1–O2	176.8(2)	O6–Co4–N7	94.51(19)
Na2A–O10	2.365(6)	O8–Co1–O2	84.85(17)	O12–Co4–N7	94.35(19)
Na2A–O4	2.447(6)	O1–Co1–N3	88.5(2)	O11–Co4–N7	93.02(18)
Na2A–O15A#	2.785(18)	O7–Co1–N3	89.2(2)	O5–Co4–N7	91.20(18)
Na2A–O15B#	2.540(15)	O8–Co1–N3	93.28(19)	O6–Co4–O14A	87.4(5)
Na2B-O17	2.244(10)	O2–Co1–N3	93.5(2)	O6–Co4–O14B	79.0(5)
Na2B-O10	2.267(5)	O1–Co1–N4	89.0(3)	O12–Co4–O14A	76.6(4)
Na2B-O4	2.397(5)	O7–Co1–N4	88.7(2)	O12–Co4–O14B	87.9(4)
Na2B-O5	2.397(5)	O8–Co1–N4	89.2(2)	O12–Co4–N7	94.35(19)
Na2B-O11	2.402(5)	O2–Co1–N4	88.6(2)	O11–Co4–N7	93.02(18)
Na2B-O15B#	2.803(14)	N3–Co1–N4	176.9(2)	O11–Co4–O14A	85.2(5)
Na2B-O15A#	2.989(18)	O13–Co2–N1	176.42(17)	O11–Co4–O14B	93.5(5)
Na2B-O16B	2.993(16)	O13–Co2–O4	101.89(16)	O5–Co4–O14A	97.8(4)
Na2B-O16A	3.151(14)	N1–Co2–O4	75.20(15)	O5–Co4–O14B	86.6(4)
		O13–Co2–O3	107.49(16)	N7–Co4–O14A	170.8(4)
		N1–Co2–O3	75.82(15)	N7–Co4–O14B	173.1(4)

symmetry operation #: 1-x, 1-y, 1-z.

Table S7. BVS analysis for $[Co_8Na_4(L)_4(OH)_2(CO_3)_2(py)_{10}](BF_4)_2$ (**2**). Same method employed as for complex **1** (see above).

	r	r0 Co(II)	V Co(II)	r0 Co(III)	V Co(III)
Co1–O1	1.856	1.685	0.62992	1.637	0.55327955
Co1–O7	1.871	1.685	0.604894	1.637	0.53129788
Co1–O8	1.889	1.685	0.576171	1.637	0.50606959
Co1–O2	1.893	1.685	0.569975	1.637	0.50062804
Co1–N3	1.957	1.65	0.436167	1.75	0.57151791
Co1–N4	1.965	1.65	0.426838	1.75	0.5592934
			3.243965		3.22208637

	r	r0 Co(II)	V Co(II)	r0 Co(III)	V Co(III)
Co2–O13	1.954	1.685	0.483344	1.637	0.42453673
Co2–N1	2.039	1.65	0.349465	1.75	0.45791071
Co2–O4	2.117	1.685	0.311123	1.637	0.27326936
Co2–O3	2.116	1.685	0.311965	1.637	0.27400893
Co2–O15A	2.185	1.685	0.25889	1.637	0.22739173
Co2–N5	2.178	1.65	0.240021	1.75	0.31450454
			1.954808		1.97162199

	r	r0 Co(II)	V Co(II)	r0 Co(III)	V Co(III)
Co3–O13	1.949	1.685	0.48992	1.637	0.43031265
Co3–N2	2.044	1.65	0.344774	1.75	0.45176435
Co3–O10	2.138	1.685	0.293956	1.637	0.25819141
Co3–O9	2.137	1.685	0.294752	1.637	0.25889017
Co3–O15A	2.158	1.685	0.278489	1.637	0.24460561
Co3–N6	2.163	1.65	0.249952	1.75	0.32751671
			1.951843		1.9712809

	r	r0 Co(II)	V Co(II)	r0 Co(III)	V Co(III)
Co4–O6	1.974	1.685	0.457911	1.637	0.40219799
Co4–O12	1.982	1.685	0.448116	1.637	0.39359515
Co4–O11	2.046	1.685	0.376938	1.637	0.33107663
Co4–O5	2.049	1.685	0.373894	1.637	0.32840308
Co4–N7	2.104	1.65	0.293163	1.75	0.38413672
Co4–O14A	2.088	1.685	0.336489	1.637	0.29554951
			2.28651		2.13495908

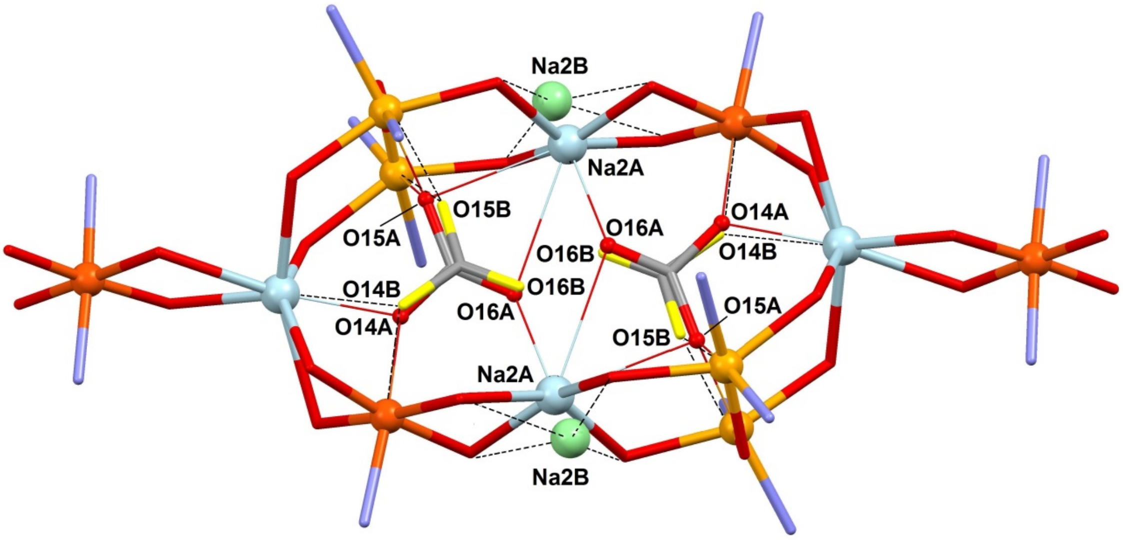

Figure S7. Core of complex **2**, showing the positions of all the atoms in both disordered forms. Atoms that vary their position in going from one form (A) to the other (B) are labelled. The colour scheme of the unaltered atoms are the same as in Fig. 5, bottom.

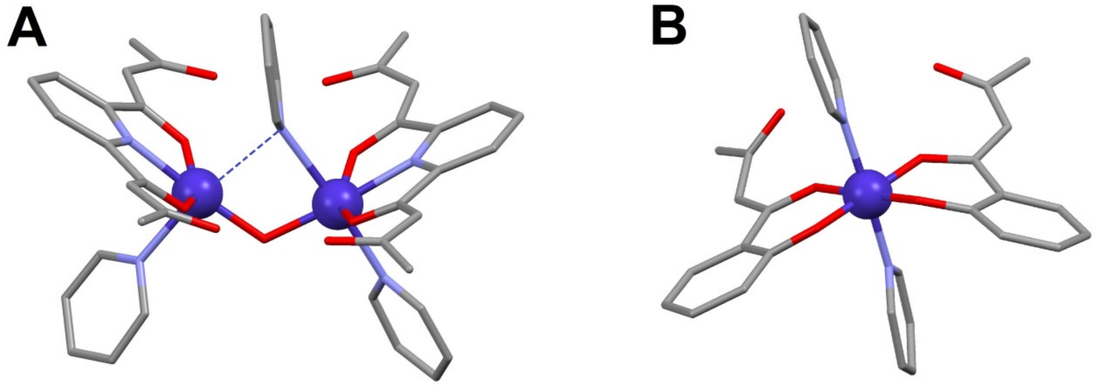

Figure S8. Structural models of **1**, from truncation of the full cluster cation $[Co_4(OH)(L)_2(py)_7]^+$ after optimization of the nuclear positions (see text). A) The fragment related to the Co(III) distal metals have been removed. B) Relevant fragment to calculate the interaction between axial pyridine and the distal Co(III) metal. Both fragments have a total charge of -1.

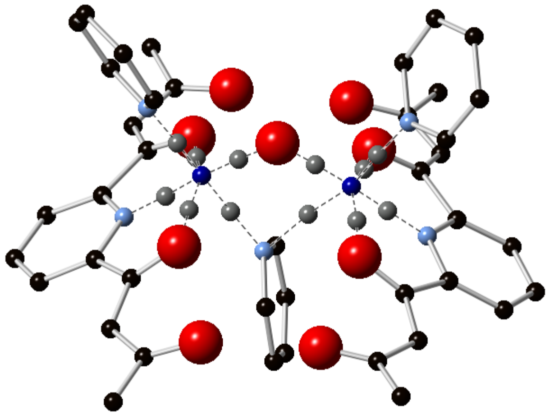

Figure S9. Bond critical points (gray balls) around atoms Co2 and Co2A of complex **1**, calculated with the experimental, truncated structure employed for DFT calculations (see text)..

Table S8. Electron density (in atomic units) at the bond critical points of Figure S9:

	Co2	Co2A
Co-N bridge	$3.75\ 10^{-2}$	$1.75\ 10^{-2}$
Co-N1	$7.34\ 10^{-2}$	$7.36\ 10^{-2}$
Co-N2 top	$5.85\ 10^{-2}$	$5.85\ 10^{-2}$
Co-O bridge	$9.00\ 10^{-2}$	$9.06\ 10^{-2}$
Co-O1	$6.23\ 10^{-2}$	$6.24\ 10^{-2}$
Co-O2	$6.51\ 10^{-2}$	$6.54\ 10^{-2}$

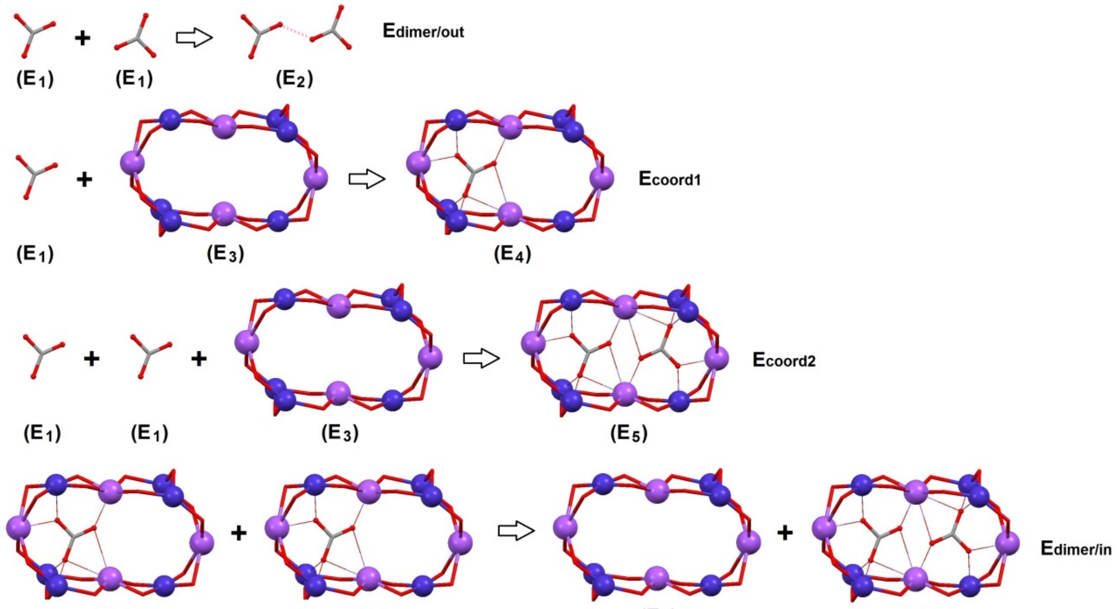

Figure S10. Scheme of the processes studied theoretically (see text). The energies of the models labelled E_1, E_2, E_3, E_4 and E_5 have been calculated by DFT. These models are only schemes of the actual systems treated, which contain all atoms (see caption Fig. 6).
